# On Adulteration of Sulphate of Quinine

**Published:** 1853-03

**Authors:** 


### MATERIA MEDICA AND THERAPEUTICS.

*On Adulteration of Sulphate of Quinine.* By Dr. Moll.—The excessive price of the true cinchona, the calisaya of Bolivia, has led to the substitution of many inferior kinds, chiefly remarkable for their containing large proportions of *quinidine.* In consequence of their lower price they have obtained admission to the quinine manufactories in large quantities, and much of the sulphate now produced is depreciated by the addition of quinidine. This substance differs from the sulphate of quinine by its greater specific gravity and less flocculent crystallization, and it is much more soluble than it is in water and alcohol. The addition of both *cinchonine* and *quinidine* may be detected by means of ether; for while cinchonine is almost insoluble in this substance, quinidine is so in a far less degree than is quinine, inasmuch as sixty drops of ether and twenty of ammonia will dissolve ten grains of quinine and only one grain of quinidine. On the addition of these quantities of sulphuric ether and liq. ammonia, to ten grains of quinine, with ten drops of dilute sulphuric acid, and fifteen of water, all will remain dissolved, unless cinchonine, or more than ten per cent. of quinidine be present, the mechanical impurities only appearing at the surface. If 10 per cent. of quinidine be present in the ethereal solution, it will soon crystallize on the surface of the ether. Traces of this substance can be yet more certainly discovered if ether saturated with quinidine be employed, when all that exists in the suspected salt will remain insoluble. If the powder contain cinchonine or more than 10 per cent. quinidine, it will remain undissolved at the line of demarcation of the two fluids. If it be quinidine, it is soluble in additional ether, which cinchonine is not.

To establish the purity of quinine, we must also assure ourselves of the absence of *inorganic* substances, by calcination in platina, or by a solution of the salt in alcohol. Sulphate and carbonate of lime, magnesia, &c., remain undissolved, while boracic acid, though soluble, betrays itself by its blue flame on conflagration. The absence of *organic* substances, as salicine, sugar, starch, stearic acid, is known by the colorless solution which takes place in concentrated sulphuric acid. The presence of *ammoniacal salts* is revealed by the odor which ensues on the addition of caustic alkali.—*Brit. and For. Med. Chir. Rev. from Revue Medico-Chirurgicale.*

